# Novel frame shift mutation in *ERCC6* leads to a severe form of Cockayne syndrome with postnatal growth failure and early death: A case report and brief literature review

**DOI:** 10.1097/MD.0000000000011636

**Published:** 2018-08-17

**Authors:** Yao Kou, Mohammad Shboul, Zhihao Wang, Qasem Shersheer, Zhaojie Lyu, Peirong Liu, Xiaodong Zhao, Jing Tian

**Affiliations:** a Key laboratory of Resource Biology and Biotechnology in Western China, College of Life Sciences, Northwest University, China; b Department of Medical Laboratory Sciences, Jordan University of Science and Technology; c AL-Zarqa Government Hospital-Pediatric Department, Jordan; d Shanghai Center for Systems Biomedicine, State Key Laboratory on Oncogene and Bio-ID Center, Shanghai Jiao Tong University, China.

**Keywords:** Cockayne syndrome, excision repair cross-complementing group 6, nucleotide excision repair, truncated mutation

## Abstract

**Introduction:**

Cockayne syndrome (CS) is a rare multisystemic autosomal recessive disease. The primary manifestations of which are developmental delay, neurological impairment, abnormal skin sensitivity to sunlight and unique facial appearance as sunken eyes, large ears, and thin large nose. The disorders of the nucleotide excision repair system significantly are caused by mutations of Excision repair cross-complementing group 6 (*ERCC6*) and Excision repair cross-complementing group 8 (*ERCC8*) genes, and the *ERCC6* gene mutations are present in approximately 65% of cases.

**Case presentation:**

Here we described a girl in a consanguineous Jordanian family with abnormal facial appearance and postnatal growth delay. She was not able to gain weight. Her condition deteriorated progressively and she developed difficulty of swallowing even to water. The patient was diagnosed as CS based on her facial appearance and neurologic dysfunction. The patient was examined at 3 years old, and died at 4 years old.

**Conclusion:**

Genetic analysis and sequencing revealed homozygosity for a novel frame shift mutation c.2911_2915del5ins9 (p.Lys971TryfsX14) in the ERCC6. The mutation is predicted to delete 5 nucleotides and add 9 nucleotides with a premature termination, resulting in approximately 34% length reduction of the wild-type transcript. The multisystem malformations of CS are clinically heterogeneous. The frame shift mutation of *ERCC6* found in this patient is a novel one, which caused postnatal growth failure and early death. Our findings indicate truncated mutation in CS lead to more severe CS phenotype and add to the genotype–phenotype correlations in CS.

## Abstract 2

Supplemental Digital Content is available in the text

## 1 Introduction

Cockayne syndrome (CS; MIM 133540, 216400) is a rare autosomal recessive neurodegenerative disorder, which was first reported in 1936 by Sir Edward A.^[1]^ CS is characterized by cachexia bird-like, mental retardation, microcephaly, cataracts, photosensitivity, and growth failure. As a progressive disorder, the symptoms of CS aggravate with time. According to its clinical phenotype, CS can be divided into 3 types: Type I CS (CSI) is the classical CS, which the fetus develops normally during the prenatal stage. The abnormalities usually appear before 1 year old. It is manifested by neural function deficiency, skin photosensitivity, deep sunken eyes, and development retardation. The condition worsens with age, most patients die before the age of 20. Type II CS (CSII) is a kind of severe type, mainly exhibits growth defects at birth, with the severe impairments of neurological development. Most patients die before 6 to 7 years of age. Type III CS (CSIII) is mild form with normal growth during prenatal and postnatal stages. The symptoms of CS appear progressively in childhood and adulthood.^[2–4]^ Meanwhile, there are numerous other CS subtypes including Cerebro-oculo-facio-skeletal syndrome 1 (COFS1; OMIM 214150) and UV–sensitive syndrome 1 (UVSS1; OMIM 600630). COFS is the most severe type of CS which can be considered as a prenatal form of CS; UVSS is a very mild type of CS which can be reorganized by cutaneous photosensitivity alone without any neurological involvement or growth defect.

CS has been found to be caused by mutations in 2 genes, *ERCC6* (also known as *CSB*, OMIM 609413) and excision repair cross-complementing group 8 (*ERCC8*, also known as *CSA*, OMIM 609412). As a pathogenic gene for approximately 65% CS,^[3]^*ERCC6* gene encodes a 168-kDa protein with 1493 amino acids. ERCC6 protein contains an acidic domain, a glycine-rich region, 2 putative nuclear localized signal sequences, and 7 characteristic helicase ATPase domains.^[5]^ Belonging to the SWI2/SNF2 family which usually involved in chromatin remodeling, transcription and DNA repair, ERCC6 has been implicated in various DNA repair transcription processes,^[6,7]^ however, the detailed mechanisms account for CS still remain poorly understood.

Here we reported a female proband from a consanguineous Jordanian family with a severe CS phenotype when she was examined at 3 years old. The proband was dead at 4 years old. A novel frame shift mutation c.2911_2915del5ins9 (p.Lys971TryfsX14) in the ERCC6 was identified which resulted in a frameshift and a premature termination, leading to approximately 34% length reduction of ERCC6 protein. This case further contributes to the phenotype spectrum seen in CS, and gives evidence to phenotype–genotype correlations.

## 2 Case report

**Figure 1:**
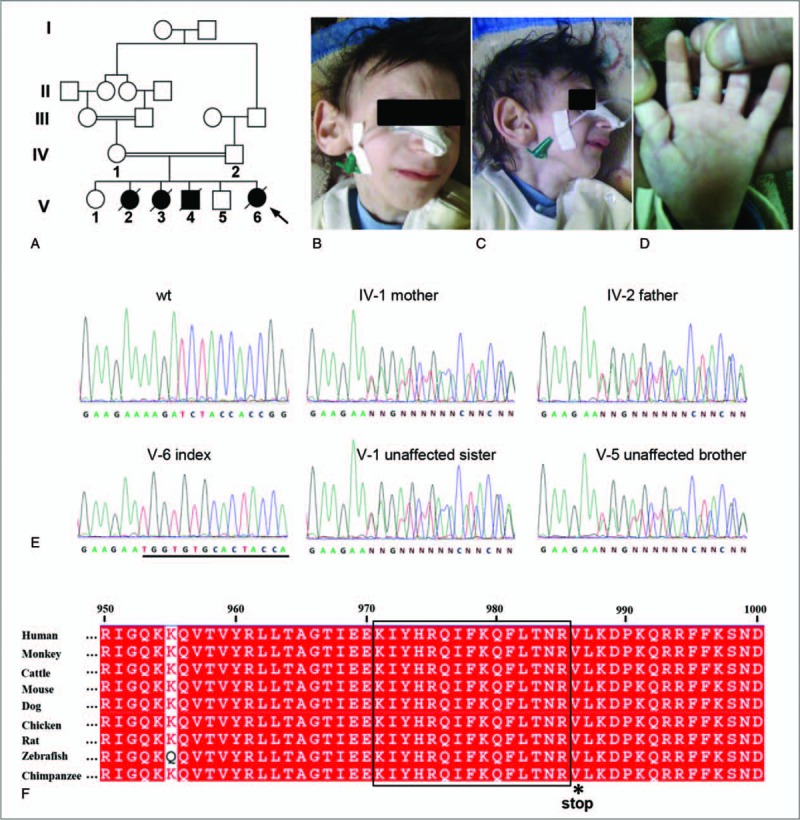
Phenotypic characteristics and mutation identification of investigated patient. (A) The pedigree of the studied family, the proband was indicated by the black arrow. The patient exhibited Mickey Mouse appearance (B and C) and sclerotic epiphyses of the fingers in her hand (D). (E) Sanger sequencing chromatographs showing a homozygous AAGAT>TGGTGTGCA mutation in the patient and heterozygous for the parents and 2 unaffected siblings compare to the normal people. (F) This mutation occurs in a highly conserved region of ERCC6, a frameshift from K971 to R985 is marked by a black rectangle, a premature stop codon at amino acid 986 is highlighted with a star (∗).

The index reported here with Cockayne syndrome was born at a consanguineous Jordanian family (Fig. 1A). In the prenatal period, the fetus did not gain weight at 28 weeks of gestation. The girl was born at term, weighing 2.6 kg, looked normal from facial appearance but her feet were stretched all the time, no history of admission to NICU (neonatal intensive care unit). During her postnatal period, the girl started to develop abnormal facial appearance like microcephaly, beaked nose, micrognathia, high palate, large ear and sunken eyes which gave the patient a Mickey Mouse appearance (Fig. 1B and C). She had postnatal growth failure which was not able to gain weight. The girl's condition deteriorated progressively and she developed difficulty of swallowing even to water. The girl was totally dependent on nasogastric tube 3 times per day. She had delayed social interaction with others. She also exhibited short stature, long limbs with joint contractures, large hands and feet, kyphosis, scoliosis, thickened calvariea, sclerotic epiphyses of the fingers^[3,8–17]^ (Fig. 1D, Table 1). At 3 years old, the girl's mother came to hospital to seek medical advice for her. Brain CT scan of the patient showed there was large symmetrical and bilateral intracranial calcification in frontal par ventricular and occipital lesions, widening cerebral sulci, large occipital subarachnoid space seems to be communicating with the 4th ventricle, and hypoplasia of cerebellum. Mental retardation and sensorineural deafness indicated the neurologic abnormal of the patient. Ophthalmologic findings indicated the index had microphthalmia with blepharokeratoconjunctivitis. The patient's mother did not seek medical advice early for the patient because the mother also had 2 daughters and one son with the same condition and all of them died at the very early childhood stage (but not confirmed by molecular analysis). The patient was diagnosed as CS based on her facial appearance and neurologic dysfunction at the time of examined (Table 1). The patient died at 4 years old.

**Table 1:**
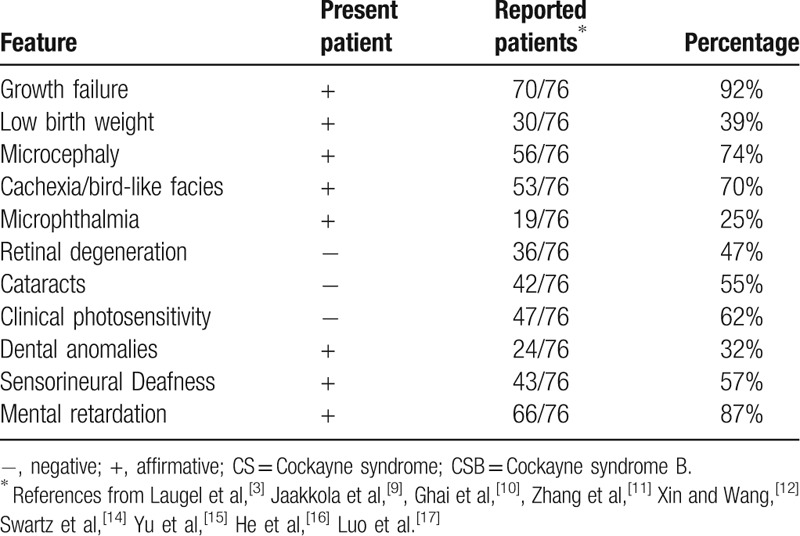
Main clinical features of CS patient in current study compared to CSB patients in the literatures.

## 3 Mutation analysis

All human studies were in accord with and approved by the Review Boards of Northwest University. Genomic DNA from saliva samples from the 5 members of the kindred (Fig. 1A: IV:1, IV:2, V:1, V:5, and V:6) were obtained after parents gave their informed consent forms and the Medical Ethics Committee of National Center for Diabetes, Endocrinology and Genetics gave its approval*. ERCC6* and *ERCC8* gene were checked by Sanger sequencing. Primer pairs for each exon and the flanking intron regions of *ERCC6* and *ERCC8* were designed using Primer3.0 (see Table S1, Supplemental Content, which demonstrates primers used for *ERCC6* and *ERCC8* genes screening). Polymerase chain reaction was used to amplify DNA segment running on Applied Biosystems PRISM 3730 Analyzer.

Sequencing analysis of *ERCC6* and *ERCC8* genes revealed a deletion/insertion AAGAT>TGGTGTGCA mutation at exon 16 of *ERCC6* gene. This c.2911_2915del5ins9 is a frameshift mutation which is previously unreported. The mutation was heterozygous for the parents and 2 unaffected siblings but homozygous for the index (Fig. 1E). This mutation occurs in a highly conserved region of ERCC6 (Fig. 1F), causes a frameshift from K971 to R985, leads to a premature stop codon at amino acid 986 (p. V986X) (Fig. 1F). This mutation was not found in 100 healthy control individuals (data not show).

## 4 Prediction protein structure analysis

We used SWISS-Model Repository (http://swissmodel.expasy.org/repository/)^[18]^ and I-TASSER (Iterative Threading ASSEmbly Refinement)^[19]^ to analyze the protein structure, conservation domain and functional domain.

**Figure 2:**
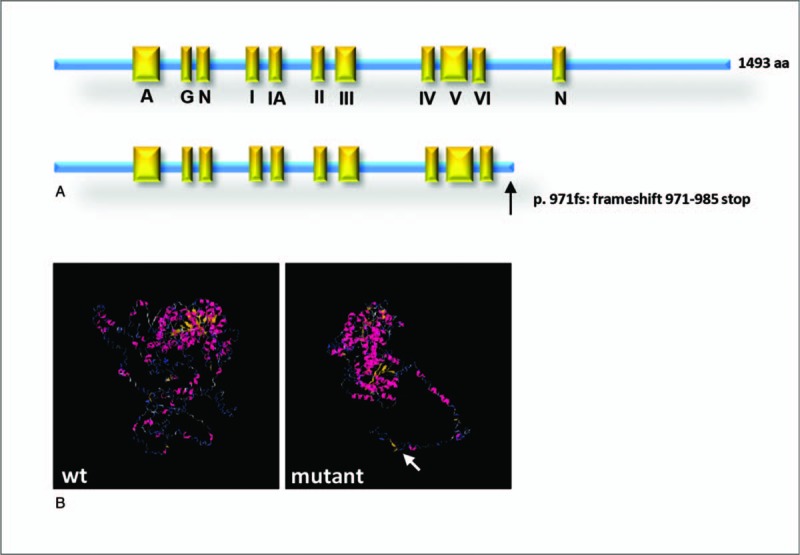
(A) Schematic view of ERCC6 domains and localization of identified truncated mutation. The black arrow indicated the mutation site (p. 971fs: frameshift 971-985 stop) (B) Predicted wild type and truncated mutation of ERCC6 protein structures. The white arrow indicated the long, opened 3’ terminal tail of mutant ERCC6 protein.

The severe truncation leads to a loss of 508 amino acids, which was 34% of full length of ERCC6 protein, including a nucleotide binding fold domain (N) and some other basic structure regions (Fig. 2A). Protein structure prediction using I-TASSER software exhibits, comparing to ERCC6 full length protein, the truncated protein cannot be folded properly with a long, opened 3’terminal tail (Fig. 2B). The 3-D structure analysis indicates reduced or abnormal protein function of ERCC6 in patient.

## 5 Discussion

Due to the significant progress in the past few years on the studies of CS, the pathogenesis of CS is more and more clear. CS is caused by impairments of the nucleotide excision repair (NER) system.^[20]^ NER is the major DNA repair process that attempts to remove DNA damage induced by ultraviolet or chemical irradiation and to keep normal replication or transcription. As a member of NER pathway, ERCC6 plays important role in DNA transcription, repair and other activities which is the process of ATP dependence.^[21]^ The ATPase domain of ERCC6 is a necessary component for ultraviolet induced DNA damage repair.^[22,23]^

Genetic analysis has defined 2 major subtypes of the DNA repair disorder of CS: CSA and CSB, which are caused by mutations of *ERCC8* and *ERCC6* respectively. CSB patients occupy two-third cases of CS, with a broad phenotype spectrum. Up to date, at least 83 mutations in more than 74 reported patients have been identified in *ERCC6* gene, including missense mutations (18.1%), nonsense mutations (30%), short insertions and deletions mutations (33.7%), splicing mutations (16.9%), and promoter mutations (1.2%) (Table S2, Supplemental Content, which demonstrates distribution of different types of mutations in *ERCC6* gene). These mutations are distributed along the whole genomic sequence and most types of mutations are represented. Among these identified mutations categories, nonsense mutations, as well as short deletion and insertion mutations are 2 major mutation types, which account for 63.7% of all the mutations. As a kind of severe or even fatal autosomal recessive neurodegenerative disorder, the prenatal diagnosis at the genetic level is necessary. The family medical history and family pedigree should be first analyzed to determine whether the fetus is at the risk for CS. The fetus genomic DNA should be screened for mutations in *ERCC6* and *ERCC8* genes. With the effective prenatal diagnosis, the incidence of CS would be reduced.

Genotype–phenotype correlation could be analyzed only if there were clearly recognizable and relatively homogeneous phenotype. The multisystem malformations of CSB are clinically heterogeneous, encompassing a wide range of clinical symptoms in types and severities, from a very severe prenatal COFS syndrome to the mildest UVSS. In an attempt to gather further insights of genotype–phenotype correlation in CSB patients, we summarized the reported CSB cases with deletion and/or insertion mutations (Indels)^[3,4,24–28]^ (Table 2). There are 20% of cases show COFS phenotype, and 48% cases exhibit CSII phenotype, only 20% CSI and 12% CSIII. There is no mildest UVSS case reported. There are only 8 homozygous mutations in total 28 Indels (28.6%), but the cases caused by homozygous mutations account for nearly half of all cases (12 out of 25 reported cases), especially in COFS, 4 out of 5 patients are caused by homozygous mutations. Short deletions or insertions in the coding part of an mRNA always results in frameshifting changes, which could lead to inappropriate or premature stop codon. In 25 Indel cases, 68% are severe types of CSB which suggests that a truncated or abnormal CSB protein could be more deterious than the completely lack of CSB protein. This might be one of the direct reasons for the more severe symptoms of CSB. In our patient, the homozygous p.Lys971TryfsX14 mutation causes a very severe CSII phenotype. The patient's condition worsened progressively and died at 3 years of age due to loss of function of ERCC6 caused by a reduced or abnormal ERCC6 protein.

**Table 2:**
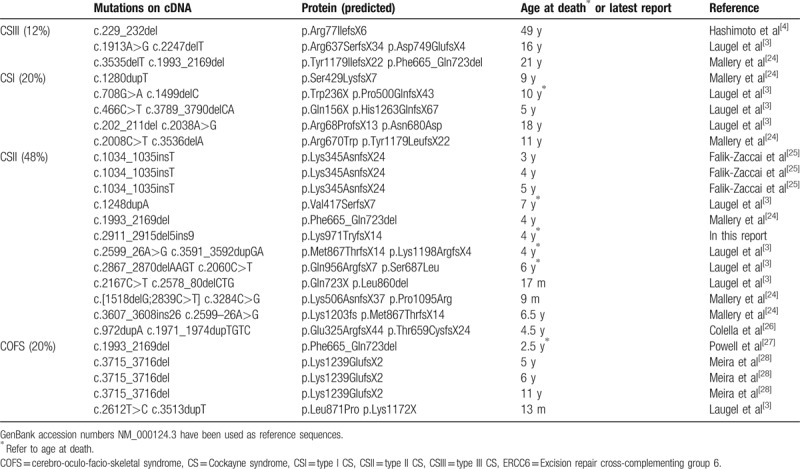
Classification of reported patients with short deletion and insertion ERCC6 mutations.

In summary, a novel homozygous mutation c.2911_2915del5ins9 (p.Lys971TryfsX14) in *ERCC6* gene was identified from a consanguineous Jordanian family. The patient exhibited severe CSII phenotype with postnatal growth failure and early death. We propose that the structurally abnormal ERCC6 protein might not only completely lose its functional activity but probably also its ability to interact with other cellular proteins. More clinical and molecular data, as well as crystal structure analysis will be needed to elucidate the complex genotype–phenotype correlations for CS mutations and to understand the functional consequences of the identified mutations.

## Acknowledgments

We are indebted to the family for kindly partaking in this study.

## Author contributions

**Conceptualization:** Jing Tian, Mohammad Shboul.

**Data curation:** Yao Kou.

**Formal analysis:** Yao Kou, Zhihao Wang.

**Funding acquisition:** Jing Tian.

**Investigation:** Qasem Shersheer.

**Resources:** Jing Tian, Mohammad Shboul.

**Supervision:** Jing Tian.

**Validation:** Zhaojie Lyu, Peirong Liu.

**Writing – original draft:** Yao Kou.

**Writing – review & editing:** Jing Tian, Xiaodong Zhao.

## Supplementary Material

Supplemental Digital Content
